# Rates of COVID-19 Among Unvaccinated Adults With Prior COVID-19

**DOI:** 10.1001/jamanetworkopen.2022.7650

**Published:** 2022-04-20

**Authors:** Jessica P. Ridgway, Samuel Tideman, Bill Wright, Ari Robicsek

**Affiliations:** 1 Department of Medicine, University of Chicago, Chicago, Illinois; 2 Providence Research Network, Renton, Washington

## Abstract

This cohort study compares rates of COVID-19 between unvaccinated adults who did and did not previously test positive for SARS-CoV-2.

## Introduction

Risk of SARS-CoV-2 reinfection among unvaccinated people with prior COVID-19 is a subject of debate.^1,2^ We performed a survival analysis in a large US population to assess the degree and duration of protection associated with natural immunity in unvaccinated individuals.

## Methods

This cohort study used data from patients tested for SARS-CoV-2 at 1300 sites of care in 6 western US states in the Providence health care system between October 1, 2020, and November 21, 2021. Patients who were unvaccinated for and had symptoms consistent with COVID-19 at the time of testing were included. Beginning 90 days after their initial SARS-CoV-2 nucleic acid amplification test (NAAT), patients were monitored for subsequent COVID-19, as determined by a positive SARS-CoV-2 NAAT result in the presence of symptoms.

We performed Cox proportional hazards regression to analyze COVID-19–free survival among patients with prior COVID-19 (positive for SARS-CoV-2 on their initial test [cases]) compared with patients who tested negative for SARS-CoV-2 on their initial test (controls), adjusting for age, sex, and race and ethnicity (based on medical record documentation). Patients were censored at their last primary care or inpatient visit during the study period (encounters in which clinicians consistently verified vaccination status using electronic medical record and outside data) or when they received a COVID-19 vaccine, died, or tested positive for SARS-CoV-2. We calculated the level of protection associated with prior COVID-19 as 1 minus the hazard ratio (HR) for COVID-19 among cases vs controls. We measured protection over time by calculating a 50-day rolling mean of the protection level and estimated 95% CIs with 1000 × bootstrap sampling. This study was approved by the Providence institutional review board, which waived the informed consent requirement because the study was considered to have minimal risk. We followed the (STROBE) reporting guideline and used R, version 4.1.2 (R Foundation for Statistical Computing) for the statistical analysis.

## Results

We identified 24 043 cases and 97 572 controls; 2762 controls (2.8%) developed COVID-19 compared with 98 cases (0.4%) (Table). The Figure shows disease-free survival among cases and controls. In the survival model, the HR among cases for developing COVID-19 was 0.15 (95% CI, 0.13-0.18); for hospitalization for COVID-19, 0.12 (95% CI, 0.08-0.18); and for COVID-19 not requiring hospitalization, 0.17 (95% CI, 0.13-0.21). Prior COVID-19 was associated with protection of 85% against any recurrent COVID-19, 88% against hospitalization for COVID-19, and 83% against COVID-19 not requiring hospitalization. Protection remained stable over the study period with no attenuation up to 9 months from initial infection.

**Table.** Demographic Characteristics of Cases and Controls^a^

Characteristic	Cases (n = 24 043)	Controls (n = 97 572)
Age, mean, (SD), y	42.0 (19.8)	37.7 (22.0)
Sex, No. (%)		
Female	13 240 (55.1)	57 055 (58.5)
Male	10 803 (44.9)	40 517 (41.5)
Race and ethnicity, No. (%)		
Asian	1052 (4.4)	3869 (4.0)
Black	975 (4.1)	4212 (4.3)
Hispanic	7261 (30.2)	15 601 (16.0)
White	11 996 (49.9)	63 114 (64.7)
Other^b^	2759 (11.5)	10 776 (11.0)
Time from enrollment to censoring, median (IQR), d	156 (117-229)	160 (122-226)
Total follow-up time, d	2 120 616	8 935 007
Patients with subsequent COVID-19, No. (%)	98 (0.4)	27625 (2.8)

^a^ Cases were defined as patients with an initial positive COVID-19 test result; controls, patients with an initial negative COVID-19 test result.

^b^ Other included American Indian or Alaska Native, Native Hawaiian or Other Pacific Islander, and unknown race and ethnicity.

**Figure.**
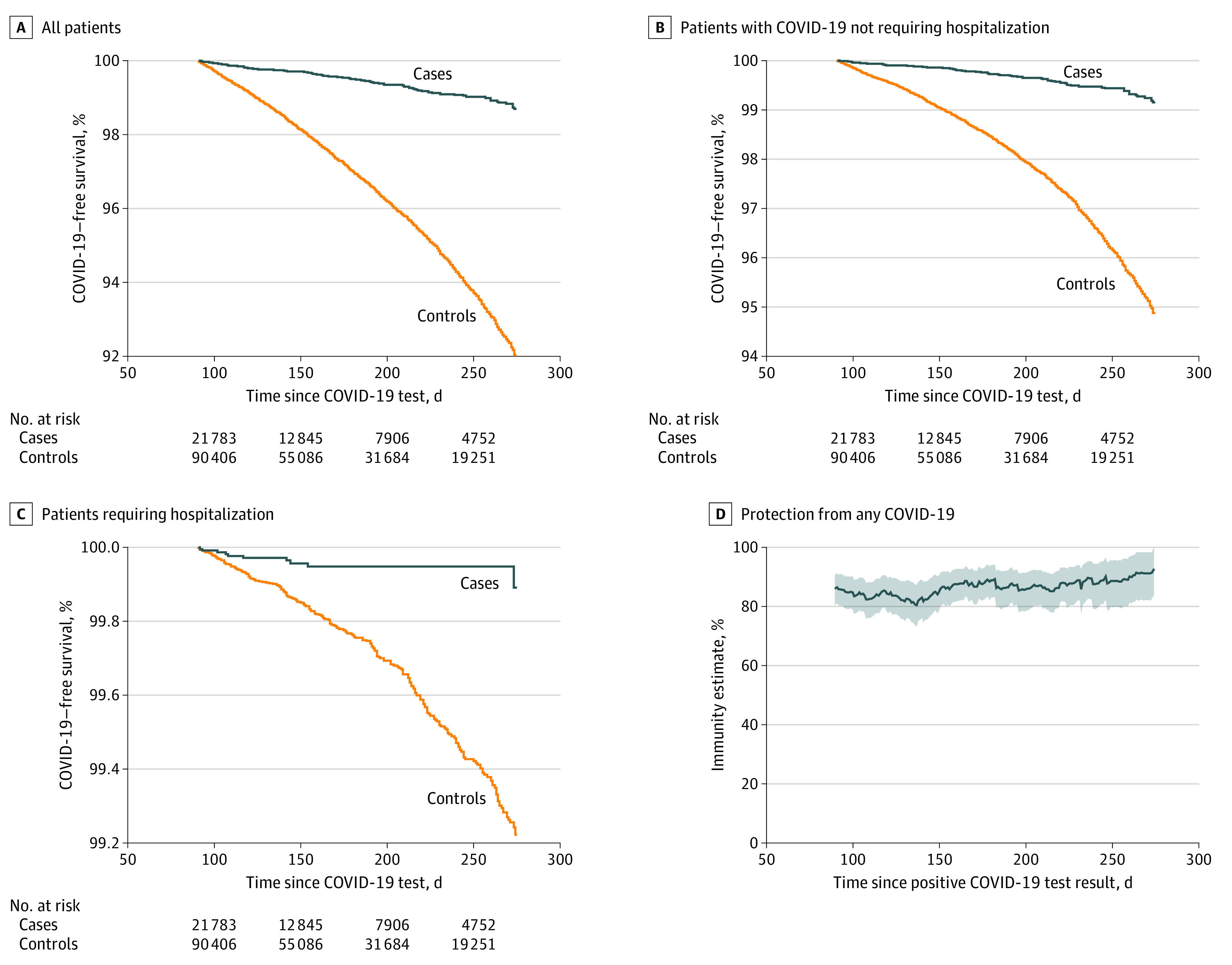
COVID-19–Free Survival Among Unvaccinated Adults With and Without Prior COVID-19 A-C, Cases were defined as patients with an initial positive COVID-19 test result; controls, patients with an initial negative COVID-19 test result. D, Protection over time was calculated as the 50-day moving mean of the protection level among cases.

## Discussion

Among 121 615 patients with more than 10 million days of follow-up, unvaccinated individuals with prior symptomatic COVID-19 had 85% lower risk of acquiring COVID-19 than unvaccinated individuals without prior COVID-19. Prior studies investigating protection against SARS-CoV-2 reinfection found similar results, with protection associated with natural immunity ranging from 80.5% to 100%.^2,3,4^ This level of protection is similar to that reported for mRNA vaccines.^5^ The findings that patients with prior COVID-19 had 88% protection against hospitalization for COVID-19 and 83% protection against COVID-19 not requiring hospitalization suggest that natural immunity was associated with similar protection against mild and severe disease. mRNA vaccines are associated with similar prolonged protection from severe COVID-19 as found in our study, although vaccine-associated protection from mild COVID-19 has been shown to wane at 6 months.^6^

Limitations include possible COVID-19 testing or vaccination at outside health care facilities, but undetected infection should have been balanced between cases and controls. Patients who have recovered from COVID-19 may behave differently from those without immunity, potentially confounding results. Strengths include large sample size, long duration of follow-up, and inclusion of only unvaccinated individuals with symptomatic COVID-19. The findings of this study may have important implications for vaccine policy and public health.
